# Risk of Amyotrophic Lateral Sclerosis in Patients With Diabetes: A Nationwide Population-Based Cohort Study

**DOI:** 10.2188/jea.JE20140176

**Published:** 2015-06-05

**Authors:** Yu Sun, Chien-Jung Lu, Rong-Chi Chen, Wen-Hsuan Hou, Chung-Yi Li

**Affiliations:** 1 Department of Neurology, En Chu Kong Hospital, Sanxia District, New Taipei City, Taiwan; 2 Master Program in Long-Term Care, College of Nursing, Taipei Medical University, Taipei, Taiwan; 3 School of Gerontology Health Management, College of Nursing, Taipei Medical University, Taipei, Taiwan; 4 Department of Physical Medicine and Rehabilitation, Taipei Medical University Hospital, Taipei, Taiwan; 5 Department and Institute of Public Health, College of Medicine, National Cheng Kung University, Tainan City, Taiwan; 6 Department of Public Health, College of Public Health, China Medical University, Taichung City, Taiwan

**Keywords:** diabetes mellitus, motor neuron disease, relative risk, cohort study

## Abstract

**Background:**

Glucose intolerance in patients with amyotrophic lateral sclerosis (ALS) has been inconsistently reported. Evidence for the association of ALS and diabetes mellitus is limited. We aimed to assess the overall and age- and sex-specific risks of ALS among patients with diabetes in Taiwan.

**Methods:**

The study cohort included 615 492 diabetic patients and 614 835 age- and sex-matched subjects as a comparison cohort, followed from 2000 to 2008. We estimated the incidence densities of ALS and calculated the relative hazard ratios (HRs) of ALS (ICD-9-CM 335.20) in relation to diabetes using a Cox proportional hazard regression model, with adjustment for potential confounders, including sex, age, geographic area, urbanization status, Charlson Comorbidity Index, frequency of medical visit, and histories of hypertension, hyperlipidemia, and chronic obstructive pulmonary disease.

**Results:**

Over a 9-year period, 255 diabetic and 201 non-diabetic subjects developed ALS, corresponding to incidence densities of 7.42 and 5.06 per 100 000 person-years, respectively. After adjustment for potential confounders, patients with diabetes experienced a significantly elevated HR of 1.35 (95% confidence interval [CI], 1.10–1.67). A higher covariate adjusted HR was noted in men (HR 1.48; 95% CI, 1.13–1.94) than in women (HR 1.17; 95% CI, 0.84–1.64), while men aged ≤65 years showed the most increased HR of 1.67 (95% CI, 1.18–2.36).

**Conclusions:**

This study demonstrated a moderate but significant association of diabetes with ALS onset, and such association is not confounded by socio-demographic characteristics or certain ALS-related co-morbidities. Further studies are warranted to examine whether the findings observed in our study can be replicated.

## INTRODUCTION

Amyotrophic lateral sclerosis (ALS) is the most common adult-onset form of motor neuron disease. Though the exact etiologies and mechanisms are still not known, ALS is considered a multifactorial disease, with multiple genetic and environmental factors causing motor neuron degeneration.^1^

Insulin resistance and glucose intolerance in patients with ALS have been inconsistently reported since the 1960s.^2^^–^^4^ However, whether there is a causal relationship between glucose homeostasis abnormalities and ALS is controversial. In the past several decades, many hypotheses have been proposed for ALS. The most commonly hypothesized etiology of ALS includes glutamate-related excitotoxicity,^5^^,^^6^ oxidative stress,^7^ and mitochondrial dysfunction.^8^ Evidence is accumulating that these aforementioned pathogeneses are also associated with the pathophysiology of diabetes and some of its complications.^9^^–^^12^ Despite the possible common pathophysiologic pathways, studies on the association between diabetes and ALS are very limited in number.^13^^,^^14^ A recent systematic review revealed that evidence for the association of ALS and diabetes mellitus was derived mainly from cross-sectional studies.^14^ To the best of our knowledge, no longitudinal study is available concerning the effects of diabetes on the subsequent risk of ALS.

The aim of this study was to examine the putative link between diabetes and risk of ALS onset. Given the rare occurrence of ALS, our investigation employed a large nationwide diabetic population in Taiwan to prospectively examine whether diabetes mellitus may increase the future risk of developing ALS in various age and sex stratifications.

## METHODS

### Data source and identification of study subjects

Data analyzed in this study were retrospectively retrieved from the claims of the National Health Insurance Research Database (NHIRD), which provides inpatient and ambulatory medical records for around 99% of Taiwanese people.^15^ We used data from the NHIRD for the ambulatory care claims, all inpatient claims, and updated registry for beneficiaries from 1997 to 2008 for this study. All National Health Insurance datasets can be interlinked with each individual personal identification number (PIN). Since the NHIRD consists of de-identified secondary data released to the public for research purposes, the study was exempt from full review by the Institutional Review Board. However, access to the NHIRD was reviewed and received ethical approvals from the National Health Research Institutes reviewing committee (No. 93126) in order to ensure appropriate use of claims data.

This is a retrospective population-based cohort study. Details of the claims data and methods of selection of patients with diabetes and comparison cohorts were described in our previous reports.^16^ Briefly, diabetic patients were considered to be diagnosed with ICD-9 code 250 or A-code A181 in the diabetic ambulatory care claims in 2000 and again within the subsequent 12 months. The first and last outpatient visits within 1 year must be >30 days apart to avoid accidental inclusion of miscoded patients. Additionally, we excluded patients who had ever been diagnosed with ALS (ICD-9-CM 335.20) between January 1, 1997, and the date of initial ambulatory care visit for diabetes treatment in 2000. The final cohort consisted of 615 492 diabetic patients. For each diabetic patient, the index date was set as the date of his/her initial ambulatory care visit for diabetes in 2000.

The comparison cohort group was identified from the registry of beneficiaries, which accumulates information including PIN, date of birth, sex, geographic area of each member’s NHI units, and dates of enrollment and withdrawal. We excluded people with claims for ambulatory care for diabetes or ALS, and selected age- and sex-matched subjects by using the frequency matching procedure. We finally selected 614 835 non-diabetic subjects. The index date for subjects in the comparison group was the first date of enrollment to the NHI. If their first date of enrollment was before January 1, 2000, the index date was set as January 1, 2000, which was the starting point of follow-up.

### End-points and covariates

**Figure 1.**
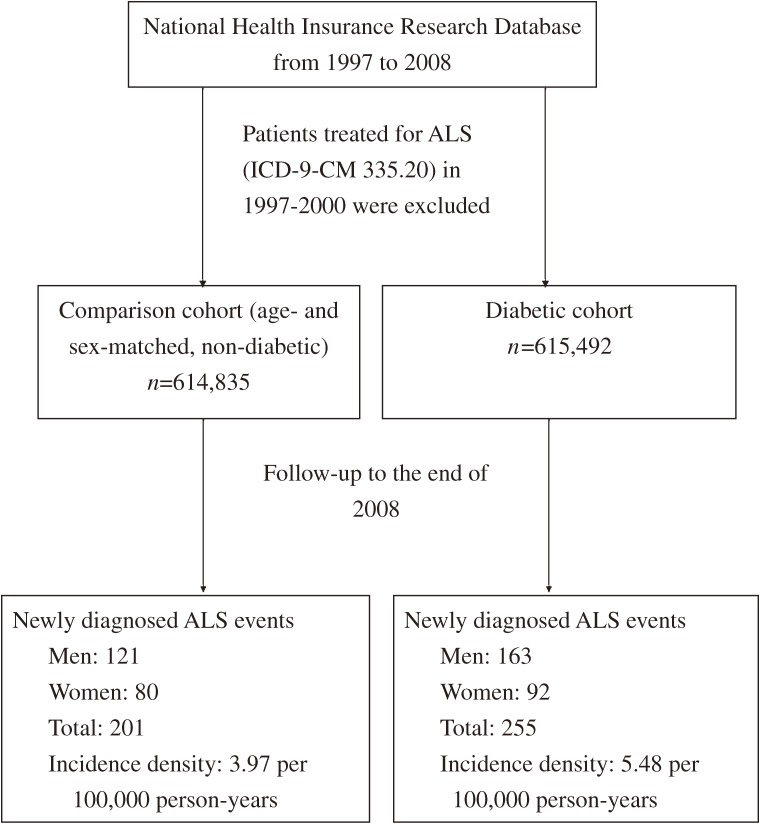
Flowchart for the study cohort enrollment and follow-up.

We used the unique PIN of each study subject in both groups and linked them to the inpatient and outpatient claims from 2000 to 2008 in order to identify the diagnoses of ALS (ICD-9-CM: 335.20), which was the end point of this study. In Taiwan, major illness/injury certificates are issued to all patients with a diagnosis of ALS. Neurologists are required to provide complete medical records, including clinical history, laboratory, imaging, and electrophysiological data, to a committee composed of a panel of expert neurologists in the application for this approval for patients. In order to avoid miscoding, we retrieved only those patients using major illness/injury certificates of this particular diagnosis. The date of encountering the clinical endpoint of interest was the first day of diagnosis either from ambulatory claims or from the primary/secondary discharge diagnosis of inpatient claims (Figure 1).

Information on a study subject’s underlying illnesses was retrieved from the inpatient and outpatient claims from January 1, 1997, to the index date of 2000. The comorbidity score was calculated using the Charlson Comorbidity Index (CCI), a scoring system for common comorbid conditions that is weighted according to mortality risk.^17^^–^^19^ We identified the potential risk factors for ALS as the confounding illnesses, including hypertension (ICD-9: 401–405), hyperlipidemia (ICD-9: 272), and chronic obstructive pulmonary disease (ICD-9: 410–414, 430–438). We adjusted for geographic area to minimize potential confounding by differential accessibility and availability of medical care.^20^ Adjustment for urbanization was to account for the possible urban-rural difference in prevalence of certain environmental factors, such as rural living and herbicide/pesticide exposure-related neurotoxins, which might be associated factors for ALS.^21^ The geographic location of each individual’s NHI unit (North, Central, South, or East) and the level of urbanization (ie, urban or rural) was categorized by the National Statistics of Regional Standard Classification.^22^ The frequency of outpatient visits for each study subjects was also adjusted to avoid the problem of potential disease surveillance bias arising from the fact that patients with diabetes are more likely than their control counterparts to seek medical care, leading to a spuriously elevated risk of ALS in diabetes.

### Statistical methods

The age- and sex-specific incidence density was first estimated with person-years as the denominator under the Poisson assumption. The study period started from the index date to occurrence of either end point, withdrawal from the NHI, or December 31, 2008, whichever date came first. Study subjects who died or withdrew from the health insurance policy were considered censored in the survival analysis. The date of censoring for those who did not encounter ALS incidence was the date on which they died, the last withdrawal from NHI, or the date of study termination (ie, December 31, 2008). We categorized age into elderly (>65 years) and otherwise. To determine the independent effects of diabetes on the risk of ALS, we used Cox proportional hazard regression models, adjusting for age, geographic area, urbanization status, CCI, and selected underlying illnesses. We also tested the interaction of age and diabetes on the risk of ALS. All statistical analyses were performed with SAS version 9.3 (SAS Institute, Cary, NC, USA). A *P* value <0.05 was considered statistically significant.

## RESULTS

The mean (standard deviation [SD]) age of patients with diabetes was 60.10 (12.73) years, and the mean age of control subjects was 60.00 (12.84) years. Patients with diabetes and non-diabetic subjects were also comparable with respect to sex, distribution of geographic area, and urbanization status. Compared to non-diabetic subjects, patients with diabetes were more likely to suffer from hypertension, hyperlipidemia, COPD, and other co-morbid conditions, with the mean (SD) CCI for diabetes and non-diabetes being 0.34 (1.29) and 0.08 (0.73), respectively (Table 1).

**Table 1.** Characteristics of the study subjects

Variables	Comparison cohort *n* = 614 835		Diabetic cohort *n* = 615 492	
	
	*n*	%	*n*	%
General Characteristics				
Age				
≤65	385 954	62.77	385 707	62.67
>65	228 881	37.23	229 785	37.33
Mean age (SD), years	60.00	12.84	60.10	12.73
Sex				
Women	319 293	51.93	319 317	51.88
Men	295 542	48.07	296 175	48.12
Geographic area				
Northern	269 224	43.79	269 901	43.85
Central	151 682	24.67	141 309	22.96
Southern	168 986	27.48	178 619	29.02
Eastern	17 938	2.92	17 944	2.92
Missing	7005	1.14	7719	1.25
Urbanization status				
Urban area	402 687	65.50	409 942	66.60
Rural area	205 143	33.37	197 831	32.14
Missing	7005	1.14	7719	1.25
Co-morbidity				
CCI				
0	590 734	96.08	527 169	85.65
1	14 018	2.28	44 192	7.18
≥2	10 083	1.64	44 131	7.17
Mean (SD)	0.08	0.73	0.34	1.29
Mean number of ambulatory visit in 2000 (SD)	18.72	18.06	31.94	21.06
Hypertension				
Yes	369 300	60.06	526 005	85.46
No	245 535	39.94	89 487	14.54
Hyperlipidemia				
Yes	228 787	37.21	438 366	71.22
No	386 048	62.79	177 126	28.78
COPD				
Yes	260 690	42.40	300 741	48.86
No	354 145	57.60	314 751	51.14

CCI, Charlson Comorbidity Index; COPD, chronic obstructive pulmonary disease; SD, standard deviation.

**Figure 2.**
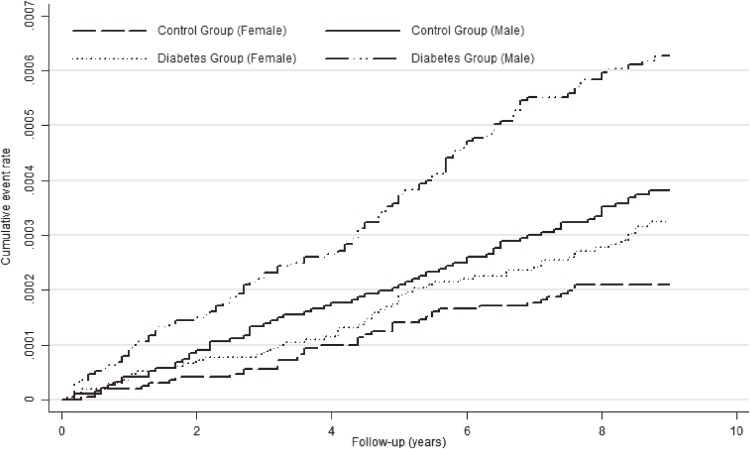
Sex-specific Kaplan-Meier survival curves for ALS in study subjects aged ≤65 years.

Over the 9 years of follow-up, ALS was diagnosed in 255 patients with diabetes and 201 individuals in the comparison cohort. For the diabetic group, the mean follow-up was 7.56 years and the total follow-up was 4 563 285 years. The respective figures for the comparison cohort were 8.23 years and 5 062 972 years. The overall incidence density of ALS in diabetics was 5.48 per 100 000 person-years, and that in controls was 3.97 per 100 000 person-years. The incidence of ALS was nearly double in men versus women for both diabetic and comparison cohorts. For diabetic patients, the incidence density of ALS per 100 000 person-years was 7.42 (95% CI, 6.33–8.65) in men and 3.75 (95% CI, 3.03–4.60) in women; in the comparison cohort, the incidence per 100 000 person-years was 5.06 (95% CI, 4.20–6.05) in men and 2.99 (95% CI, 2.37–3.83) in women (Table 2). In the comparison cohort, people aged >65 years showed a higher incidence density than younger (≤65 years) individuals in both men and women. However, the incidence density was similar in older and younger diabetic patients, regardless of gender (Table 2). Figure 2 shows sex-specific Kaplan-Meier survival curves for ALS in study subjects aged ≤65 years.

**Table 2.** Overall and age-specific incidence densities and relative hazards of ALS (ICD-9: 335.20) in the diabetes and comparison cohorts

Variables	Comparison cohort			Diabetic cohort			Crude HR (95% CI) in association with diabetic group	Adjusted HR^b^ (95% CI)^b^ in association with diabetic group
	
	Number of patients	Number of events	ID (per 100 000 patient-years) (95% CI)^a^	Number of patients	Number of events	ID (per 100 000 patient-years) (95% CI)		
Men								
≤65	190 640	69	4.24 (3.30–5.36)	190 441	111	7.34 (6.03–8.83)	1.70 (1.26–2.30)	1.67 (1.18–2.36)
>65	104 902	52	6.82 (5.10–8.95)	105 094	52	7.61 (5.68–9.98)	1.13 (0.77–1.65)	1.19 (0.78–1.84)
Total	295 542	121	5.06 (4.20–6.05)	295 535	163	7.42 (6.33–8.65)	1.45 (1.15–1.84)	1.48 (1.13–1.94)
Women								
≤65	195 314	40	2.34 (1.67–3.18)	194 714	59	3.68 (2.80–4.75)	1.55 (1.04–2.32)	1.48 (0.94–2.33)
>65	123 979	40	4.17 (2.98–5.68)	124 587	33	3.89 (2.68–5.47)	0.93 (0.59–1.47)	0.84 (0.50–1.41)
Total	319 293	80	2.99 (2.37–3.73)	319 301	92	3.75 (3.03–4.60)	1.24 (0.92–1.67)	1.17 (0.84–1.64)

Overall	614 835	201	3.97 (3.44–4.56)	615 492	255	5.48 (4.82–6.19)	1.37 (1.14–1.64)	1.35 (1.10–1.67)

ALS, amyotrophic lateral sclerosis; CI, confidence interval; HR, hazard ratio; ID, incidence density.

Inconsistency between total population and population summed for individual variable was due to missing information.

^a^Based on Poisson assumption.

^b^Based on Cox proportional hazard regression with adjustment for sex, age, geographic area, urbanization status, Charlson Comorbidity Index, frequency of medical visit, history of hypertension, hyperlipidemia and chronic obstructive pulmonary disease.

Compared to non-diabetic subjects, diabetic patients had a significantly increased risk of ALS, with an overall adjusted hazard ratio (HR) of 1.35 (95% CI, 1.10–1.67). The sex-specific adjusted HRs were 1.48 (95% CI, 1.13–1.94) for diabetic men and 1.17 (95% CI, 0.84–1.64) for diabetic women. However, such gender differences were not statistically significant (*P* = 0.29). We observed that the adjusted HR was most increased in younger (≤65 years of age) diabetic men (HR 1.67; 95% CI, 1.18–2.36). As for younger (≤65 years) diabetic women, the crude HR was 1.55 (95% CI, 1.04–2.32), but the statistical significance attenuated after adjustment for potential confounders (HR 1.48; 95% CI, 0.94–2.33). For older men and women (>65 years), the risk of ALS in the diabetic group was not significantly increased (Table 2).

## DISCUSSION

In this large population-based study over a 9-year period, we found that diabetes was associated with an increased risk of ALS onset in men, especially in those aged ≤65 years. These results were not confounded by socio-demographic characteristics or certain ALS-related co-morbidities. The loss of statistical significance in younger women after adjustment of potential risk factors might be due to the small number of events. Though there is no clear explanation for the age-related variability in the relationship between pre-morbid diabetes and subsequent risk of ALS, evidence obtained from our study suggests that attention should be paid to those age groups with a higher relative risk.

The association between ALS and glucose intolerance was first observed around 50 years ago.^23^ However, the results were not consistent, and whether abnormal glucose homeostasis is the cause or a consequence of ALS has not been fully clarified.^2^^,^^4^^,^^24^ Over the past decades, several studies explored the potential risk factors of sporadic ALS, including metabolic factors, vascular disorders, or lifestyle behaviors such as smoking and drinking.^13^^,^^25^ A case-control study investigated whether antecedent diabetes is associated with the development of ALS, but the results were inconclusive due to the small sample size.^13^ Previous studies failed to provide evidence of the putative link between diabetes and future risk of ALS.^25^ The present study is, to our knowledge, the first large population-based cohort study to provide epidemiological evidence suggesting the association between diabetes and the risk of ALS.

The cause of ALS is still not known, but it is believed to be a multifactorial and multisystem disease. One of the major hypotheses of pathogenesis is excitotoxic stimulation due to accumulation of glutamate, which is associated with both diabetes and ALS.^5^^,^^26^ Functional activity of neurotransmitter receptors and their sensitivity to regulation are altered in diabetes.^26^ Increased oxidative stress is another common theory of pathogenesis for both ALS and diabetes. Convincing evidence showed that oxidative stress is a deleterious factor leading to insulin resistance, β-cell dysfunction, impaired glucose tolerance, and ultimately, type 2 diabetes,^10^^,^^27^ with elevated markers of oxidative stress in insulin-resistant subjects. There is also substantial evidence implicating oxidative stress as a central mechanism by which motor neuron death occurs, including elevated markers of oxidative damage in ALS patients’ spinal cord and cerebrospinal fluid and mutations in the antioxidant enzyme superoxide dismutase 1 (SOD1), causing approximately 20% of familial ALS cases.^7^ Furthermore, elevated levels of iron have been found in ALS spinal cord tissue,^28^ and this may contribute to oxidative damage via the ability of iron to generate reactive oxygen species through the Fenton reaction.

This study showed an increased risk of ALS in diabetic men aged ≤65 years. Diabetes in younger people is metabolically distinct from diabetes in the elderly. Older type 2 diabetic patients tend to be less obese and are more likely to have significantly impaired insulin secretion than middle-aged type 2 diabetic patients.^29^ The hallmark of type 2 diabetes in the young, as in most adults, is insulin resistance. Defects in insulin receptor function, the insulin receptor-signal transduction pathway, glucose transport and phosphorylation, glycogen synthesis, and glucose oxidation contribute to muscle insulin resistance.^30^ Whether diabetes- or insulin resistance-related oxidative stress may increase the risk of motor neuron death in young men warrants further investigation.

A hypothesis of mitochondrial defect in substrate oxidation in disorders of insulin resistance, with a decrease in mitochondrial density and mitochondrial copy number, has been proposed.^9^ As a consequence of fuel oxidation, mitochondria generate considerable amounts of reactive oxygen species, and these radicals are implicated in the pathophysiology of diabetes and its complications in multiple organs and neurons.^11^ Mitochondria, playing crucial roles in excitotoxicity and cell apoptosis, are thought to be an early target in ALS pathogenesis and contribute to disease progression.^8^^,^^31^ Morphological and functional defects in mitochondria were found in both human patients and ALS mice overexpressing mutant SOD1. Mutant SOD1 was preferentially associated with mitochondria and subsequently impaired mitochondrial function, which may further cause the axonopathy in ALS.^8^ Such information reveals the link between the mitochondrial dysfunction of insulin resistance individual and the pathogenesis of motor neuron.

The strengths of this study are the use of a large, nationally representative population-based cohort, with little possibility of recall and selection bias and little likelihood of non-response and loss to follow-up of cohort members. In addition, the adjustment of geographic area, urbanization status, and frequency of outpatient visits for each study subject might have reduced area-related confounding factors and disease surveillance bias. However, several limitations should also be noted. First, diagnoses of diabetes, ALS, or any other comorbid medical conditions that are completely dependent upon ICD codes may be less accurate than those obtained through a standardized procedure. This is a major limitation of this study compared to studies using standardized examinations of patients. However, the National Health Insurance Administration (NHIA) of the Ministry of Health and Wealth conducts quarterly expert reviews of a random sample for every 50 to 100 ambulatory and inpatient claims at each hospital and clinic. Any hospital with outlier charges or outlier practice patterns, or which is suspected of malpractice, faces the risk of an audit and subsequent heavy penalties by the NHIA when discrepancies, overcharging, and malpractice are discovered.^32^ In addition, we used at least two diabetes-related diagnoses with the first and the last visits >30 days apart, which may largely reduce the likelihood of disease misclassification.^16^^,^^33^ As for the diagnosis of ALS, code 335.20 was found to be a useful tool for case ascertainment, with high sensitivity and specificity.^34^ Further, in the present study, we retrieved only those patients using major illness/injury certificates, with ALS diagnosis reconfirmed by an expert committee to avoid miscoding. The second limitation was that we were unable to differentiate between type 1 and type 2 diabetes in the present study, which may also limit specific interpretations of the study results. Third, we could not determine the severity, duration, or treatment regimens of diabetes, or smoking habit, alcohol consumption, occupation, and other socioeconomic characteristics in our study population, which might have also confounded the study results.^35^

In summary, the results of this population-based cohort study suggest an increased risk of ALS among diabetic patients, particularly men aged ≤65 years. The biological mechanisms behind the association of diabetes with the risk of ALS are, however, not understood at present. Further studies to replicate our findings in other populations, as well as to evaluate if age and sex of patients may interact with diabetes in causing the occurrence of ALS, should be conducted. In addition, although a large-scale screen for ALS in patients with diabetes may not be cost-effective, both clinicians and diabetic patients should be informed of the accumulating evidence on the relationship between diabetes and ALS so that early detection and appropriate management of ALS can be done in patients with diabetes, especially those aged ≤65 years.
